# Sub-parts per billion detection of trace volatile chemicals in human breath using Selected Ion Flow Tube Mass Spectrometry

**DOI:** 10.1186/1756-0500-1-41

**Published:** 2008-07-10

**Authors:** Brian M Ross

**Affiliations:** 1 Northern Ontario School of Medicine, Departments of Biology and Chemistry, and the Public Health Program, Lakehead University, Thunder Bay, Ontario, Canada

## Abstract

**Background:**

Selected ion flow tube mass spectrometry (SIFT-MS) allows the real time quantification of trace gases in air. Due to its tolerance of high humidity levels the technique is particularly suited to the chemical analysis of breath. The detection limit of SIFT-MS has previously reported to be approximately 5 – 10 PPBV which is insufficient for the measurement of some low abundance constituents of breath. Recent developments in the design of SIFT-MS instruments have increased the ion precursor count rates. It is, however, unclear as to how these advances will affect instrument sensitivity for breath analysis.

**Findings:**

Standard gases were prepared by adding known quantities of compounds present at zero or very low levels in breath (xylene and toluene) to either humidified bottled air or actual human breath. These were then analysed by SIFT-MS to calculate the limits of detection for each compound under conditions which mimic a single breath exhalation. For xylene and toluene the limits of detection was approximately 0.5 PPBV per 10 seconds of analysis time. Results gained using this level of sensitivity suggested the presence of low levels of the compounds indole and methylindole in human alveolar and static oral air, although further studies are necessary to confirm these findings.

**Conclusion:**

Recent advances in SIFT-MS have increased the techniques sensitivity for breath analysis into the sub PPBV range enabling the real time quantification of low level trace gases in human breath.

## Background

The measurement of volatile gases in breath for the purpose of the diagnosis, screening and monitoring of disease is an attractive proposition due to the inherently non-invasive nature of such methodology [1]. For example, the volatile biomarker nitric oxide has been successfully developed for the monitoring of airway inflammation while other putative markers for a conditions including cancer have been identified [2]. To be adopted clinically the methods used for the detection and quantification of breath biomarkers likely need to be rapid, simple to use, reproducible and able to detect the presence of disease early in its course. The latter feature can reduce mortality, morbidity, patient distress and decrease associated costs for the healthcare system [3]. As such more sensitive detection methods may offer the advantage that volatile biomarkers can be detected and acted upon at earlier stages in the disease process.

A technique which shows promise for diagnostic breath analysis is Selected Ion Flow Tube Mass Spectrometry (SIFT-MS). SIFT-MS is suited to this role due to its linear response, reproducible absoloute concentration measurements, rapid analysis and lack of interference by the major constituents of expired air, in particular water vapour [4]. SIFT-MS is a form of chemical ionization in which precursor ions (usually H_3_O^+^, NO^+ ^or O_2_^+^) are reacted with gas mixtures to produce ionized products characteristic of the volatile chemicals present [4]. The specific precursor ions used in the reaction, formed using a microwave water vapour ion source, are selected using a quadrupole mass filter. The precursor ions are then introduced into a fast flowing stream of helium and reacted with the air samples containing the trace gases of interest in a short flow tube. The ionized products along with remaining precursor ions are then quantified using a downstream quadrupole mass analyser. Once the rate constant for the reaction is known absolute concentrations of the trace gases present can be calculated without the need for calibration standards.

The sensitivity of SIFT-MS instruments has been a limiting factor since many potential biomarkers are present at concentrations in the low parts per billion by volume (PPBV) range or lower. This abundance is currently considered to be at or beneath the limits of detection for the technique, reported to be in the single digit parts per billion range for a single breath (10 second measurement time) [4-6]. The sensitivity of SIFT-MS instruments is partly dependent on the rate constant for the reaction between the precursor ion and the trace gas but also on several modifiable factors: the rate of precursor ion production, the amount of trace gas introduced in a given time, the measurement time and the background 'noise' signal produced in the absence of analyte. Recently it has been reported [7] that doubling the sampling flow rate allowed the detection of phosphine gas at concentrations of approximately 200 parts per trillion by volume (PPTV). Whether such sensitivities can be achieved for breath analysis by this modification is unclear since the authors used dry nitrogen as a carrier gas rather than the humid air mixture characteristic of human breath [7]. This difference is of importance since increasing the sampling flow rate of expired breath elevates the proportion of precursor ions reacting with the abundant water vapour thereby potentially antagonising the beneficial effect of the increased sampling rate [4]. Alternatively, a recent report suggests that optimizing the water content of the gas mixture used in the instrument's ion source can markedly elevates the rate of precursor ion generation. Using this modification the effect of enhanced precursor rates upon instrument sensitivity for the analysis of humid air mixtures has been investigated.

### SIFT-MS analysis

SIFT-MS analysis was performed using a Profile 3 instrument (Instrument Science, UK) modified to allow a variable water vapour abundance to enter the microwave source as described [8]. By this means H_3_O^+ ^count rates of approximately 2.2 × 10^6 ^counts per second (cps) were achieved while introducing ambient air in the SIFT-MS, with the total count rate of H_3_O^+ ^plus it's hydrates (H_3_O^+^.(H_2_O)_n _where n = 1, 2 or 3) being approximately 2.5 × 10^6 ^cps; typical maximal O_2_^+ ^count rates were approximately 3 × 10^6 ^cps while NO^+ ^were approximately 1.8 × 10^6 ^cps.

### Gas standards prepared with humidified bottled gas

For these experiments the compounds xylene and toluene (Sigma Aldrich, USA) were chosen since both are present in low levels in the breath of healthy non-smokers (BRoss, unpublished observations). A 5 L Tedlar bag (SKC Inc, USA) was filled and evacuated 3 times with bottled air to reduce the concentration of bag derived volatile compounds. The bag was then inflated with bottled air before followed by the introduction of 20 mL of water into the bag via the sampling valve port. The bag was then heated to 40°C for 30 minutes to produce humidified air which was used in subsequent experiments. A measured quantity (260 mL) of the humidified air was then transferred to empty 0.5 L Tedlar bags by means of a heated syringe. Up to 500 μL of a commercially available gas standard containing approximately 5 parts per million by volume xylene and toluene in helium (Standard and Technical Gases, United Kingdom) was then introduced into the 0.5 L bags using 50, 250 or 500 μl capacity gas syringes (Fisher Scientific, Canada) via a septum to produce known concentrations of each gas. After further incubation at 40°C for 15 minutes to allow mixing, the standards were introduced into the SIFT-MS by negative pressure via a transfer line heated to 65°C at a flow rate of 0.21 Torr L s^-1^. Measurements were made by opening the inlet valve and waiting 10 seconds before quantifying gas levels over the period 10 to 20 seconds.

**Figure 1:**
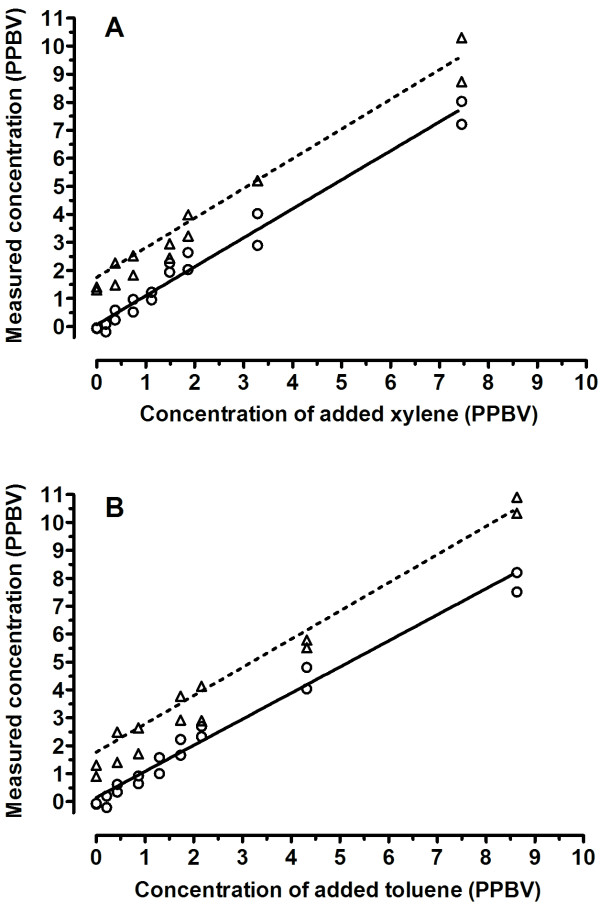
**Detection of xylene and toluene added to humidified air and human breath**. Xylene (A) or toluene (B) vapour were added to either humidified bottled air (circles) or human breath (triangles) contained in Tedlar bags to produce a range of concentrations in duplicate. The standards were introduced into the sampling line of the SIFT-MS and after a delay of 10 seconds xylene and toluene levels were measured by reaction with the H_3_O^+ ^precursor for a period of 10 seconds. These values were then plotted against the calculated standard concentrations. The best fit linear regression line is shown for both bottled air (solid line) and human breath (dashed line).

The water content of the gas samples prepared using humidified bottled air (5.8 ± 0.1% (mean ± SD) was similar to that of human breath. The concentration of xylene and toluene (measured by their reaction with the H_3_O^+ ^precursor as previously described in detail [9]) in humidified bottled air prior to introduction of these chemicals was not differentiable from the instrument background i.e. that derived from the count rate determined in the absence of sampled air. Using a 10 second sampling period both xylene and toluene could be differentiated from background at concentrations of approximately 500 PPTV (Figure 1) with instrument blanks (sampling line closed) being approximately 0.5 cps for both compounds, while 500 PPTV resulted in approximately 2 cps product ions. Increasing concentrations of both compounds produced a linear rise in measured concentration over the concentration investigated (approximately 0 – 8 PPBV).

### Gas standards prepared using human breath

A breath sample was obtained from a 40 year old male in good health followed by the construction of gas standards using a similar method as for bottled air with the exception that no humidification was required. Specifically, the subject exhaled into a 3 L Tedlar bag and 260 mL was transferred into 0.5 L Tedlar bags, followed by the addition of known quantities of the 5 PPMV gas standard in helium by means of a gas syringe. The subject gave informed consent for the procedure. Detectable concentrations of xylene (product ion m/z 107, *k *= 2.3 × 10^-9 ^cm^3 ^s^-1^) and toluene (product ion m/z 93, *k *= 2.1 × 10^-9 ^cm^3 ^s^-1^) product ions, measured using the H_3_O^+ ^precursor, were present in the absence of added standards (approximately 1 PPBV for xylene and toluene). These ions could well be derived from other compounds, however levels measured were significantly above instrument backgrounds (levels recorded in the absence of sampled air) and of similar concentrations (approximately 1 – 2 PPBV) when xylene or toluene levels were measured using reaction with the NO^+ ^or O_2_^+ ^precursors (xylene: product ion m/z 106, *k = *1.4 × 10^-9 ^cm^3 ^s^-1^; toluene: product ion m/z 92, *k *= 1.4 × 10^-9 ^cm^3 ^s^-1^). It should be noted that the proton bound dimer of ethanol has the same m/z as the product of the reaction between toluene and H_3_O^+^. As such, in the presence of high ethanol concentrations ethanol may contribute to the apparent toluene signal making the use of the other precursor ions advisable for the analysis of this compound. It is presently unclear as to whether these xylene and toluene are compounds produced metabolically or are due to ambient levels of these gases which were present in the environment at similar concentrations. Using a 10 second sampling period, 500 PPTV exogenous concentrations of both compounds were differentiable from the exhaled breath levels (Figure 1).

### Measurement of trace malodorous compounds in human breath

In order to further investigate the ability of SIFT-MS to detect compounds present at concentrations of approximately 1 PPBV or less alveolar breath analysis was performed using three human subjects (a 40 year old female, a 41 year old male and a 26 year old female all of whom gave informed consent). Alveolar breath and static oral air were sampled as described [10]. Briefly, to sample alveolar breath the subject expired via a heated stainless steel tube into which was inserted the SIFT-MS sampling line so that a small fraction of the flow was drawn into the instrument using negative pressure at a flow rate of 0.21 Torr L s^-1^. For oral air the stainless steel sampling tube was sealed at one end and an adapter attached at the other end which allows a piece of PTFE tubing (length 10 cm, OD 1/4", ID 3/8") to be attached. The subject was asked to close their mouth and breathe though their nose for 3 minutes to concentrate volatile compounds in the mouth. A piece of tubing is then inserted 2.5 – 5 cm into a nearly closed mouth. The subject is asked to not touch any mouth surface with the tube, and must not blow into, or inhale through, the tubing. Levels of the test compounds were measured using the H_3_O^+ ^precursor by their following product ions and utilising the following rate constants: hydrogen sulphide: m/z 35, *k *= 2.0 × 10^-9 ^cm^3 ^s^-1^; methylmercaptan: m/z 49, *k *= 2.5 × 10^-9 ^cm^3 ^s^-1^; indole: m/z 118 and 136, *k *= 3.3 × 10^-9 ^cm^3 ^s^-1^; and methylindole: m/z 132 and 150, *k *= 3.3 × 10^-9 ^cm^3 ^s^-1 ^[10-12]. As expected [10] (Table 1) oral air contained significant levels of the sulphur compounds hydrogen sulphide and methylmercaptan while levels in alveolar air were very low. For methylmercaptan the actual alveolar concentrations of the gas may be lower given that the reaction of H_3_O^+ ^with the ^18^O isotopologue of ethanol will result in the production of the same product ion as that with methylmercaptan. As such approximately 0.6% of the ethanol concentration would contribute to the apparent methylmercaptan level measured using the above procedure e.g. 1 PPMV ethanol would contribute 6 PPB to the methylmercaptan concentration. Given the ubiquity of ethanol in both laboratory and clinical settings this mimicking effect of the compound should always be taken into account. The enhanced sensitivity of the SIFT-MS also allowed the detection ion product signatures of the compounds indole and methylindole. The presence of these compounds in human breath was supported by the generation of product ions of m/z 117 and 131 at rates approximately 2 cps above background for the reaction of human breath with O_2_^+^, which are consistent with products generated by charge transfer reactions with indole and methylindole respectively [12]. Such rates of product ion generation equate with similar concentration levels of the compounds with that detected using H_3_O^+^. The possibility that these ion products could be due to isopotopologues must be considered although significant generation of ions one or two mass units lower that the putative product ions for methylindole and indole was not apparent in the subjects studied. Nevertheless, as the sensitivity SIFT-MS increases the possibility that the presence of isopotopologues will become a significant factor in any particular measurement is also increased. In addition, the introduction of humidified lab air possessing a similar water content to that of human breath (6% v/v) did not change the apparent ambient levels of these compounds, suggesting that the product ions concerned are not due to a hydrate of a reaction product derived from another compound. The presented data therefore are suggestive of both methylindole and indole being present in human breath alveolar breath at low levels. Both compounds are characteristic of faecal odour and their detection in breath at low levels may derive from either the gut or from an oral bacterial source. Further investigation is however required to conclusively determine the existence of the indoles in human breath, particularly as the construction of accurate spectra is made difficult due to their apparent low abundance.

**Table 1:** Levels of trace volatile compounds in alveolar and oral air.

**Sample**	**Compartment**	**Indole**	**Methylindole**	**Hydrogen Sulphide**	**Methylmercaptan**
Ambient	NA	0.4	0	0.4	4
Subject 1	Alveolar	1.7	0.4	10	10
	Oral	2.4	0.8	48	71
Subject 2	Alveolar	2.2	0.4	9.2	9.4
	Oral	3.2	0.9	23	44
Subject 3	Alveolar	1.1	0.9	11	4.3
	Oral	1.7	1.2	41	38

Levels of indole, methylindole (all isomers), hydrogen sulphide and methylmercaptan were measured in ambient air, or in a single alveolar breath or static oral air (approximately 10 second measurement duration) of 3 healthy human subjects by SIFT-MS using characteristic products ions of the reaction with H_3_O^+ ^precursors. Values are in PPBV. NA: not applicable.

### Limits of detection and quantification

The presented data suggest that recent advances in the design of the ion source intrinsic to SIFT-MS instruments can be utilized to achieve significant sensitivity gains for breath trace gas analysis. The rate of precursor ion generation reported in this and another recent study [8] are in the 2 – 3 million counts per second range for H_3_O^+^, a rate approaching the 10 million counts per second generated by a related technique, proton transfer mass spectrometry (PTR-MS) [13]. The ion generation method used by SIFT-MS allows the selection of the alternative NO^+ ^or O_2_^+ ^precursor ions to increase the chemical resolution of SIFT-MS compared to PTR-MS, while avoiding the quantification errors encountered by PTR-MS occurring due to the variably elevated ion energies typical of the technique [14]. By optimising the water vapour content of the ion source gas mixture the precursor count rates were sufficient to allow the detection of analyte concentrations of approximately 500 PPTV using the H_3_O^+ ^precursor. This was achieved using humidified air samples similar to human breath with a sampling time typical of a single expiration (10 seconds).

The determination of the limit of detection (LOD) can also be estimated using various parameters associated with the SIFT-MS analysis [7], these being the sensitivity of the measurement (how many product ions are produced for a given concentration of analyte in a particular time) and the instrument background counts at the product m/z, that is the counts produced in the absence of analyte. For the compounds measured in this study sensitivity was in the range 2 – 4 cps per PPBV, while the background count rates for the product(s) ions were in the range 0.3 to 3 counts per second. The estimated LOD for 10 second measurements were calculated as 170, 130, 300, 440, 320 and 350 PPTV for indole, methylindole, hydrogen sulphide, methylmercaptan, xylene and toluene respectively. For the latter two compounds such computed values are in agreement with empirically derived limits of detection (see Figure 1). It is also possible to calculate the limit of quantification (LOQ), the concentration which can be determined with a particular level of precision. For a standard deviation of 20% using a 10 second measurement time the LOQs are 0.8, 0.7, 1.0, 1.3, 1.4 and 1.5 PPBV for the presumed analysis of indole, methylindole, hydrogen sulphide, methylmercaptan, xylene and toluene respectively. As such the presented data indicate that the modified instrument used in this study can produce reasonably precise measurements of each gas present at a concentration of approximately 1 PPBV. Clearly the technique is capable of lower LOD and LOQ when longer measurement times are used, but this will necessarily result in an inability to quantify trace gases present at these concentrations in a single expiration. The use of a breath storage device e.g. Tedlar bags, can allow longer measurement times to be used although this complicates the sampling methodology and can be the source of artefact.

## Conclusion

In conclusion, advances in ion source design have resulted in significant improvements in the sensitivity of the SIFT-MS procedure resulting in the potential for additional biomarkers to be readily investigated. As the ion generation process becomes better understood it is likely that additional improvements in the rate of precursor ion generation will be achieved with a concomitant enhancement of sensitivity. It should be recognized, however, that background count rates also put a limit on the trace gas concentration that can be detected in a given time period. Background counts are influenced by a variety of factors including electrical noise in the detector, impurities in the carrier helium gas, the presence of isotopologues and hydrates of other compounds, and by the production of low abundance ions in the source which possess the same m/z ratio as the reaction product of the compound of interest [15]. The two latter factors may ultimately limit the detection sensitivity of SIFT-MS for specific trace gases rather than the rate of precursor ion generation. Optimisation of the ion source and/or the upstream mass filter to reduce the introduction of unwanted ions into the flow tube may, however, alleviate the problem of unwanted low abundance source ions. Nevertheless, for many trace gases sub-PPBV detection in single breath samples by SIFT-MS is readily achievable with the likelihood of further sensitivity enhancements in the future.

## Competing interests

The author declares that they have no competing interests.

## Authors' contributions

All experimental work and production of the manuscript was by BMR.
